# Numerical-Experimental Study of the Consolidation Phenomenon in the Selective Laser Melting Process with a Thermo-Fluidic Coupled Model

**DOI:** 10.3390/ma11081414

**Published:** 2018-08-12

**Authors:** Francisco Cordovilla, Ángel García-Beltrán, Miguel Garzón, Diego A. Muñoz, José L. Ocaña

**Affiliations:** 1 UPM Laser Centre, E.T.S. Ingenieros Industriales, Universidad Politécnica de Madrid, C/José Gutiérrez Abascal 2., 28006 Madrid, Spain; agarcia@etsii.upm.es (A.G.-B.); jlocana@etsii.upm.es (J.L.O.); 2 PM-Tec Engineering S.A.S., Portos Sabana 80, Bodega 78, Cota, Cundinamarca 250017, Colombia; mgarzon@pm-tec.co; 3 Optimización Matemática de Procesos ÓPTIMO, Centro de Ciencia Básica, Universidad Pontiﬁcia Bolivariana, Circular 1, 70-01, Medellín 050031, Colombia; amunoz@pm-tec.co

**Keywords:** Selective Laser Melting, thermo fluidic, phase change, consolidation, Arbitrary Lagrangean–Eulerian Method, metallic powder

## Abstract

One of the main limiting factors for a widespread industrial use of the Selective Laser Melting Process it its lack of productivity, which restricts the use of this technology just for high added-value components. Typically, the thickness of the metallic powder that is used lies on the scale of micrometers. The use of a layer up to one millimeter would be necessarily associated to a dramatic increase of productivity. Nevertheless, when the layer thickness increases, the complexity of consolidation phenomena makes the process difficult to be governed. The present work proposes a 3D finite element thermo-coupled model to study the evolution from the metallic powder to the final consolidated material, analyzing specifically the movements and loads of the melt pool, and defining the behavior of some critical thermophysical properties as a function of temperature and the phase of the material. This model uses advanced numerical tools such as the Arbitrary Lagrangean–Eulerian formulation and the Automatic Remeshing technique. A series of experiments have been carried out, using a high thickness powder layer, allowing for a deeper understanding of the consolidation phenomena and providing a reference to compare the results of the numerical calculations.

## 1. Introduction

In the Selective Laser Melting Process (SLM), the objective, in an industrial context, is to manufacture continuous solid components layer by layer. It uses a laser beam to consolidate the metallic powder at each of the steps in the growth of the workpiece [1]. The layer-by-laser philosophy of production theoretically eliminates the geometrical complexity of the part as a restriction for the manufacturing process. This circumstance provides the additive manufacturing technologies with large advantages to face geometrically complicated designs and a high flexible demand, in comparison with conventional techniques [2].

As is well known, productivity is one of the main limiting factors for a wide use of the additive manufacturing technologies [3], restricting their industrial application to only high added value parts, such as aeronautical parts [4] or medical implants [5].

The use of high thickness powder layers in the SLM process can contribute to improve productivity by reducing the number of steps for a given height of the desired component. There are multiple works, of theoretical or experimental nature, where the thickness of the powder layer and the diameter of the laser beam lies on the microscale. In reference [6], a layer of 30 µm is used to carry out an experimental parametrization of the process. Reference [7] calculates the evolution of the temperature during the SLM process considering a beam diameter of several tens of microns. If the thickness of the layer is turned into a scale ranging from several hundred microns up to 1 mm, and, the laser beam is adjusted to a diameter of several mm, the productivity of the process necessarily experiences a dramatic increase.

Although the relation between the size of the powder layer and the beam diameter with the productivity is extremely clear, there is neither significant research nor industrial usage of large beams and thicknesses for the SLM process. It can be associated with the more complex behavior of the melt pool, when a larger amount of material is involved, which makes the process more difficult to be governed.

Although most of the relevant phenomena in the consolidation of the material are present with small bed thicknesses, such as limited diffusivity associated to poor particle contact, phase changes, and, for the liquid metal, gradients of surface tension associated with Marangoni convection, or even recoil pressure; in the case of large thicknesses these factors strongly influence the size and shape of the melt pool leading to a relatively high degree of curvature of the geometry of the consolidated material.

There are different approaches to study the consolidation of the material during the SLM process. The work of [8], highlights very important aspects of the consolidation, such as the evolution of the material throughout several phases, powder, liquid and solid. The relevance of considering the evolution of the melt pool, with explicit use of the fluidic properties of the liquid metal, such as dynamic viscosity or surface tension, is emphasized. However, the use of a discrete particle model forces the representation of the domain to be at the scale of the metallic particles (several microns) and makes it difficult, from a computational point of view, to project the behavior of the melt pool on the final dimension of the consolidated material in a real process.

A totally different approach is carried out on the models based on the activation of layers of finite elements when they are reached by the laser beam, reproducing in this way the manufacturing of the part layer by laser [9]. These models depart from the final design of the part that is going to be manufactured, study the evolution of the temperature, and, in some cases, estimate the residual stresses in the bulk of consolidated material. No analysis of the phase change or the melt pool is performed. While this approach can provide interesting results, at little computational cost, associated to processes with a very low layer thickness, where the movements of the melt pool are very small, in the case of normal or high thickness of the powder bed, the simulation cannot depart from the final design of the part since, in these cases, the final shape of the consolidated material cannot be predicted with only thermal or mechanical stress considerations. For instance, in reference [10], it can be seen that the initial powder has given rise, as a consequence of the SLM process, to a drop-like profile in the cross section of the consolidated material. It can only be due to the surface tension when the metal was in liquid state. 

The present work proposes the use of a coupled thermo-fluidic model in the frame of a moving mesh, in combination with experimental test of the process with a powder bed of 1 mm thickness. When a large thickness of the powder bed is considered, there is such an amount of liquid metal submitted to the effect of the surface tension that the differences between the original flat domain representing the powder bed and the arising droplet forces the domain not only to move but also to recursively experience re-meshing. The droplet formation process happens in a time scale of milliseconds. The model considers the evolution of the material during the different phases, powder, liquid and solid, associating some critical thermo-physical properties, such as thermal conductivity, dynamic viscosity or surface tension, with the temperature and the phase of the material, making specific considerations to describe phase change. The concatenation of the thermo-fluidic properties to describe two different phase changes, powder to liquid and liquid to solid, must be adapted to the characteristics of each process in particular. Sigmoid functions are proposed to describe the evolution of the thermo-physical properties during the different states of the matter. The use of this explicit formulation for the phase change introduces an innovative way of dealing with it, which does not have high computational requirements. The application of the finite element method on domains representing the powder bed and the substrate, makes the study of the consolidation happen at a scale where the final macroscopic dimensions of the material can be predicted, thus favoring, in this way, its application to real industrial situations. The so-called Arbitrary Lagrangean–Eulerian formulation [11] is used to allow the movement of the mesh to be performed, which, along with some specific numerical tools, like Automatic Remeshing techniques, enables the movements of the melt pool to be fully represented. A series of experimental tests was carried out using a 1 mm thickness powder bed. These tests were used, in the first place, to understand some critical aspects associated to the use of large thicknesses. Finally, theoretical and experimental cross sections were compared, showing a good degree of agreement.

## 2. Materials and Methods 

The AISI 316L has been chosen as working material. This election has its basis on the wide use of this kind of steel in those fields where the additive manufacturing technologies have the maximum potential of application [12]. The AISI 316 steel offers high mechanical resistance, good corrosion behavior, and, of interest for medical applications, good biocompatibility properties [13]. The combination of these characteristics of the material with the capability of additive manufacturing to manufacture very complex geometries, made to measure in some cases, make of high interest the use of powders of AISI 316L and understanding its behavior in the selective laser melting process.

The commercial powder METCO MetcoClad™ 316L-SI has been used to carry out the real tests. The dominating spherical shape of its particles and its granulometric size distribution, facilitate the development of theoretical hypotheses to adjust the thermo-physical properties in the modelling. Table 1 shows the main characteristics of the commercial powder used.

### 2.1. Constitutive Equations and Its Implications on the Modeling

#### 2.1.1. Absorption Coefficient of the Metal Powder

**Figure 1:**
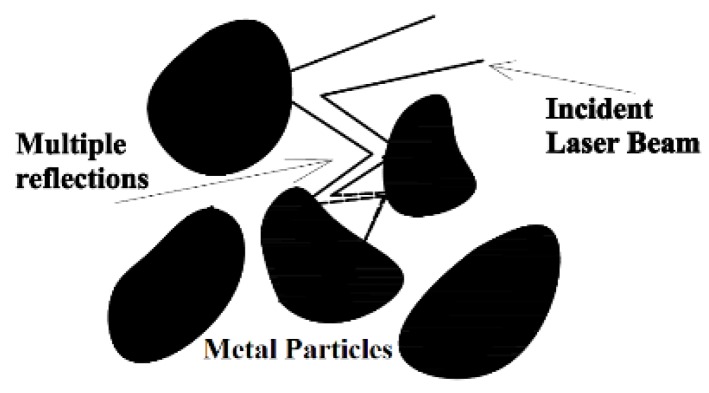
Illustration of the way in which the radiation reflected from a given particle may impact on the surrounding ones, in a multiple reflection process, resulting in a high absorption of the radiation supplied by the laser beam.

When the laser beam hits the metallic powder, part of the electromagnetic radiation is absorbed leading to an elevation of the temperature in the interaction area. During the time in which the powder is heated but not having experienced melting yet, that part of the radiation that is reflected from a given particle may impact on one of the surrounding ones, which, in turn, will behave in the same way absorbing part of the radiation and reflecting the remaining. The repetition of this process among adjacent particles (see Figure 1) leads to a higher overall absorption coefficient, especially, if compared with the situation of a laser beam irradiating a flat surface where the reflected radiation escapes from the material.

The total absorption, *A_T_*, can be characterized by the addition of the radiation absorbed by each particle *A_i_* plus the effect of the reflected radiation on each particle, when it impacts on the surrounding ones, as the product of the reflection coefficient of the first one *R_i_* by the respective absorption coefficient corresponding to the particle which receives this reflection *A_i+1_*. According to the Fresnel Equations from the electromagnetic theory [14], the absorption coefficient of radiation when it impacts on the surface of a conducive media depends on the kind of polarization of the radiation, its wavelength, the optical properties of the material (complex refraction index) and the angle of incidence between the propagation direction of the radiation and the normal vector of the irradiated surface, *θ*. While most of the indicated variables can be fixed for a given process configuration, and the complex refraction index of the material for the process wavelength can be taken from [15], the incidence angle of the laser radiation on each particle is a random variable, whose value can vary between 0 and π/2 rad. Since there is no reason to consider any value of the defined range as the most likely, this uncertainty can be solved by taking the average absorption coefficient from all the values of the range, A($\theta_{\pi /2}^{0}$). In this way *A_i+1_ = A_i_ =* A($\theta_{\pi /2}^{0}$). Additionally, not all radiation reflected from a given particle is trapped by the surrounding ones, and it can be released definitely from the bulk of powder. This phenomenon can be described by a coefficient to retain radiation within the bulk of powder, *η*, which can be related with the thickness of it, *h*. Like this, the multiple reflection process can be described mathematically by a series, as Equation (1):

(1) $$A_{T}=A(\theta_{\pi /2}^{0})+\eta \left(h\right)R\left(\theta_{\pi /2}^{0}\right)A\left(\theta_{\pi /2}^{0}\right)+\eta {\left(h\right)}^{2}R^{2}\left(\theta_{\pi /2}^{0}\right)A\left(\theta_{\pi /2}^{0}\right)+\;\eta {\left(h\right)}^{3}R^{3}\left(\theta_{\pi /2}^{0}\right)A\left(\theta_{\pi /2}^{0}\right)+\ldots$$

Equation (1) corresponds to a convergent series that can be expressed by a general term as Equation (2):

(2) $$A_{T}=\frac{A\left(\theta_{\pi /2}^{0}\right)\;}{1-\eta \left(h\right)R\left(\theta_{\pi /2}^{0}\right)}$$

#### 2.1.2. Thermo-Fluidic Coupling

Once the energy from the laser source has been absorbed by the powder, its temperature quickly increases. At the beginning, the short contact surface of the particles limits thermal diffusivity and the melting of the powder occurs just in the proximities of the laser-powder interaction zone. Once the liquid has been formed, the heat diffusion and wettability conditions improve, and the melt pool spreads beyond the limits of the interaction area [16]. The first phase change that takes places happens, therefore, for that part of the powder that becomes liquid, and the subsequent phase change will happen with the solidification of that liquid to form a consolidated solid. Both transitions, powder to liquid and liquid to solid, are driven by the thermal cycle imposed by the scanning of the laser source (fast heating and fast cooling respectively), although, each of them takes places under specific circumstances. The material which experiences melting during the heating stage evolves from being disaggregated particles with very low cohesion force among them and no continuous surface (each particle has its own one), to a continuous medium where exists friction among molecules characterized by the dynamic viscosity of the liquid metal, and, at the surface, cohesive forces associated to the surface tension of the liquid. The shape of the melt pool is configured, therefore, by the combined effect of the own weight of the melt pool (hydrostatic pressure), the influence of dynamic viscosity and surface tension. This combination of loads leads, as will be seen with the experimental and numerical results, to a geometry of the melt pool tending to acquire drop shape.

The solidification of the liquid, during the cooling stage, takes place by the increase of the cohesive forces among molecules of the material during the consolidation phenomena. In this way, the shape acquired by the melt pool tends to be maintained despite the disappearing of the effect of the balance between the surface tension and the hydrostatic pressure. 

**Figure 2:**
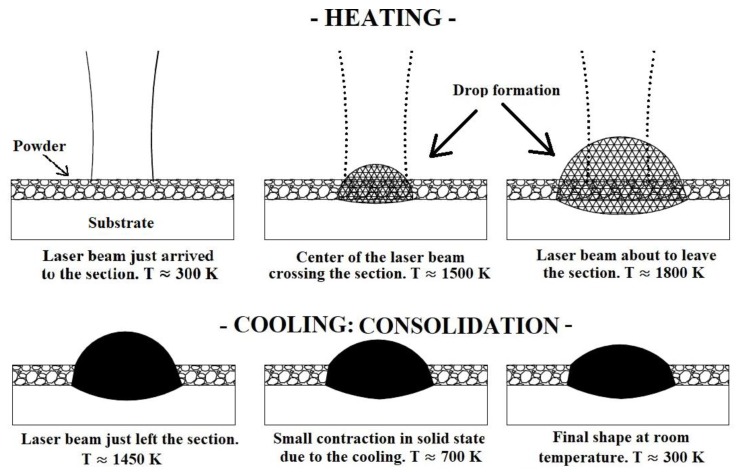
Illustrative representation of the consolidation of the material during the traversing of a given cross section by the laser beam.

Figure 2 schematically represents the traversing of the laser beam across a given section for different temperatures, T, in order to illustrate the preceding ideas. The final height of the consolidated material, as well as its drop shape are the consequence of several physics phenomena. In the first place, the poor thermal conductivity of the powder makes the energy supplied by the laser beam concentrate intensely around the laser-powder interaction area. It makes that in that region the melting point is exceeded, not only by the powder and substrate which are strictly under the laser beam, but also by the powder around it. It makes that, in the end, the volume submitted to melting is larger than just the volume of powder strictly under the laser beam. Once the volume of heated powder becomes liquid, its surface tension forces it to evolve form a volume with the shape of a rectangle in the powder be to a taller drop of liquid metal, whose height is dependent mainly of the time that the material evolves in liquid state. Additionally, given the low amount of energy needed to melt isolated particles, those of them around the melt pool are wetted by it and consequently melted, being incorporated into the melt pool, contributing significantly to the amount of liquid material. The concentration of energy under the laser-powder interaction area must also allow for melting the substrate to ensure a proper adhesion of the consolidated material to it. If the melting of the substrate does not happen, the melt pool does not penetrate it, and, although a consolidated ribbon might be formed, it is not welded to the substrate. During the melting of the powder, the air contained initially in the pours is released, making the density of the domains increase. Again, during the cooling of the material some effect of shrinkage happens, which is also reflected in the evolution of density. This effect during cooling is also considered in Figure 2.

The thermal part of the calculations is governed by the heat conduction equation in a fluidic media, Equation (3);

(3) $$\rho C_{p}\frac{\partial T}{\partial t}+\rho C_{p}u\cdot \nabla T=\nabla \left(\kappa \nabla T\right)+Q.$$

In Equation (3) the term *Q* represents any volumetric energy exchange that takes places in the studied bulk material. In this context, the latent heat of the phase changes and the metallurgical transformations are the inputs for that term. Equation (4) introduces the laser source as a surface boundary condition:

(4) $$-n\cdot \left(-\kappa \nabla T\right)=Q_{b}$$

In Equation (4) *Q_b_* represents the irradiance of the laser beam.

The fluidic evolution of the melt pool, when the material is in liquid state, is governed by the Navier–Stokes equation, as Equation (5) indicates:

(5) $$\rho \frac{\partial u}{\partial t}=\nabla \left[-pI+\mu \nabla u+\mu {\left(\nabla u\right)}^{T}\right]+\rho gz$$

In Equation (5), the last term, *ρgz*, includes the weight of the liquid as a load in the balance of forces. *ρ* is the density of the material, *g* is the gravity constant and *z* is the height under the surface of the fluid. The tangential force at the surface of the fluid *F_t_* because of the surface tension *σ* is described by Equation (6):

(6) $$-\nabla \sigma +\sigma \left(\nabla \cdot n\right)-p_{ext}n=n\cdot F_{t}.$$

In Equation (6) the term *p_ext_* includes the effect of the external pressure (the atmospheric pressure at room temperature) on the surface of the fluid.

**Figure 3:**
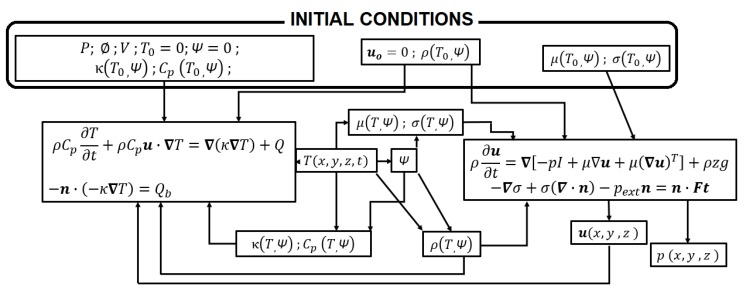
Flux diagram of the constitutive equations, boundary conditions and initial conditions of the thermo-fluidic coupling.

Figure 3 shows a flux diagram of the thermo-fluidic coupling. It happens, therefore, due to the phase and temperature dependence of some critical properties of the matter, such as dynamic viscosity, *µ*, surface tension, *σ*, and density, *ρ*. The specific capability, *C_p_*, and thermal conductivity, *κ*, which are associated exclusively with the thermal balance, also show phase and temperature dependence. For numerical purposes, a variable to identify the phase of the matter is defined, *Ψ* (Phase Variable), its value is set to 1 if the material has exceeded the melting point, and 0, if it has never happened up to the analyzed instant. 

Figure 3 shows the coupling between the thermal calculations associated to the heat conduction equation and the laser beam irradiance *Q_b_* as a surface boundary condition (block to the left) with the fluidic calculations by means of the Navier–Stokes equations submitted to surface tension, σ, and the external pressure, *P_ext_*, at the boundaries of the domain (block to the right) [17]. The top block in Figure 3 contains the initial conditions with the process parameters included. Some of them, such as the power of the laser *P*, the beam diameter *ø* (a Gaussian beam is considered), the scanning speed of the laser *V*, the heat capability *C_p_* and thermal conductivity *κ* are associated with the thermal calculations. Other properties, such as dynamic viscosity *µ* and surface tension *σ*, participate only in the fluidic calculations. In the case of density of the material, *ρ*, it influences the thermal balance since thermal diffusivity *χ* is obtained by Equation (7):

(7) $$\chi =\frac{\kappa }{\rho C_{p}}$$

and density also appears in the Navier–Stokes Equation. The velocity field of the domain, ***u***, represents the three spatial components of the speed of any point of the domain. At the beginning it is set to zero, since there is no movement of the particles in the bulk of powder. On the one hand, the motion of the fluid affects the thermal balance; on the other hand, it is the main output of the fluidic calculations. It allows for determining the movement of the fluid, and therefore, the evolution of its geometry. At the first step of calculation, the properties of the material derive from the initial temperature, *T_0_*, and initial phase, *Ψ = 0*, since the material has not experienced melting yet. The effective thermo-fluidic coupling is driven, as is implicit in Figure 3, by the thermal cycle, and therefore, by the heat calculations. Once the initial step has been taken, the rising of the temperature determines the turning of the affected points as *Ψ = 1* (the material has molten in these points). The temperature and phase dependence of dynamic viscosity and surface tension regulate, in turn, the flowing of it, associated to the heating, and, at the same time, the evolution of density, from the low apparent density of the powder to the higher density of the fluid, is able to represent the releasing of the interstitial air of the powder, and the appearance of the liquid as a continuum medium. 

During the cooling stage, the use of phase-dependent thermo-fluidic properties, along with the phase variable, *Ψ*, determines if the material must cool, either, as unmolten powder or as a rigid solid.

The theoretical basis for the consolidation of the material has been established: the driving force to turn the powder into liquid is the thermal cycle associated with thermo-fluidic properties dependent on the temperature and the phase of the matter. This dependence also determines the phenomena during cooling, giving rise to a rigid solid in those regions where the melting point has been exceeded. In this context two, challenges arise for the modelling work. From a physics point of view, the behavior of the thermo-fluidic properties must be adjusted considering the requirements of temperature and phase dependence. From the numerical point of view, the distinction between molten and unmolten region by means of a discrete variable, *Ψ*, has to be implemented on the frame of a moving mesh, capable of representing the movement of the melt pool.

#### 2.1.3. Numerical Tools to Carry Out the Thermo-Fluidic Coupling

The main goal of the thermo-fluidic approach is the calculation of the shape acquired by the liquid material under the effect of the surface tension, gradient of the surface tension, the hydrostatic pressure, the variations of dynamic viscosity and density, as well as maintaining that complex shape during the solidification of the material. It has some important implications for the numerical calculations.

Figure 2 in the previous section illustrates the cross section of a drop formation during the scanning of the laser beam. It can be seen that, starting from a flat contour representing the powder bed, the domain has to evolve to a convex contour several times higher than the original thickness of the powder bed.

The Arbitrary Lagrangean–Eulerian formulation of the Finite Element Method [11] is able to deal with the movement of the mesh and can be used to adapt it to the deformation imposed by the fluidic loads. The basis of this numerical methodology lies in the use of different coordinate systems to have a reference of the movement of the mesh. While the Eulerian part of the calculation uses a fixed reference system, the Lagrangean part uses a reference system associated with the points of the material which moves with them.

Nevertheless, the motion capability of the nodes of the mesh is not enough to represent high complex geometries. The movement of the nodes of a given element with different directions may lead to it having a high level of distortion. 

According to the general theory of the Finite Element Method [18], a high degree of distortion of the elements negatively affects the accuracy of the calculations and may lead to the impossibility of performing the convergence of the solution. It happens due to the fact that the values of the analyzed function are calculated, in the interior of each element, by interpolating the values of the nodes. If the aspect ratio of the element is too high, the result of that interpolation is wrong. 

**Figure 4:**
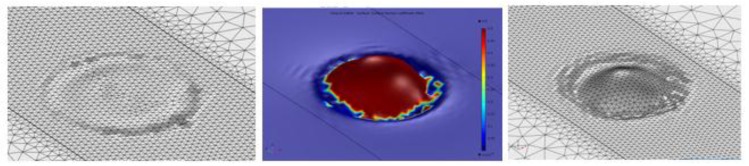
Process of an Automatic Remeshing. Departing from the initial mesh (**left**), the drop formation starts (**center**), and, when a high value of distortion in any element is reached, a new mesh is built, based on the deformed geometry (**right**).

The use of the Automatic Remeshing technique allows the problem of the distortion of the elements to be managed. It is a feature directly available in the software used for the simulations; COMSOL Multiphysics^TM^. In this way, the distortion is calculated internally by the program as a positive dimensionless number for each element. The value of this number for an undistorted element is 0. It can rise and, even descend, following the geometrical evolution of the element. When the distortion of any element reaches a critical value, the calculations are stopped, and a new mesh is built on the basis of the deformed geometry at the current simulation step. Figure 4 illustrates the simulation of the formation of a drop as a consequence of the melting of the powder. The initial mesh (left) resembles the flatness of the bed of powder. From that moment the simulation proceeds and the droplet appears progressively (center). At a given instant the distortion reaches the maximum permitted value. In that instant the numerical calculation stops, and a new mesh is automatically built by the program (right), if not exactly equal to the deformed geometry, optimized according to the algorithms of the program, and the initial user-defined mesh size.

The maximum value of the distortion at which a new mesh has to be done is user-defined. The procedure to adjust this value starts by attempting to carry out the simulation without limiting the distortion of the elements. The simulation finds problems in making the solution converge with a value of distortion about 20, and the calculations can no longer carry on. From this evidence it may be thought that by limiting the distortion to a very low value the convergence of the solution is guaranteed. Nevertheless, low values of distortion, such as 1–2, may be quickly reached and forced to stop and resume the simulation very frequently, with the associated wastes of time and memory. Considering the aforementioned restrictions, a maximum value of 5 has been used as a criterion to proceed with the automatic remeshing.

### 2.2. Thermo-Physical Properties for the Thermo-Fluidic Coupling

**Figure 5:**
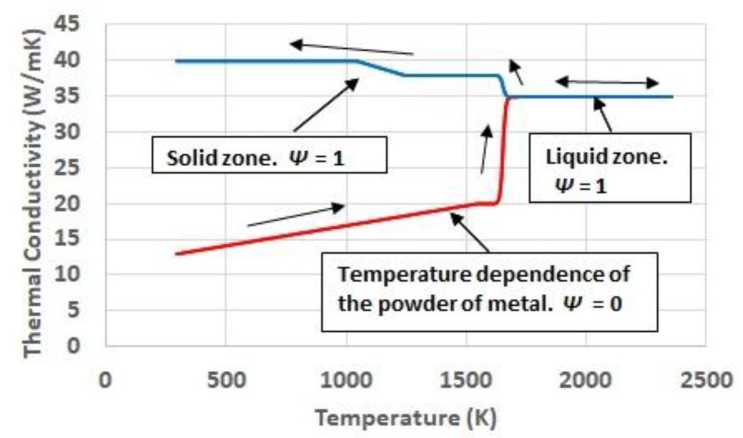
Temperature and phase dependence of thermal conductivity.

Thermal conductivity shows a high phase-dependent behavior. [19] highlights the influence of the limited contact surface among the particles or the powder bed as a limiting factor for the heat to diffuse. In the work referred to, where the AISI 316L steel is considered, the thermal conductivity of the powder at room temperature is significantly shorter than the conductivity of the same material in solid state. The present work proposes the use of different temperature functions depending on the absolute maximum temperature reached for each region of the domain. If a given region has reached, at any time, a temperature corresponding to a melting condition, its phase variable is set to, *Ψ* = 1. It determines that its cooling happens by a function different from the one during the heating, which, instead of returning to the initial conductivity of the powder, evolves along the conductivity corresponding to the cooling of the solid, considering, even, the different solid state metallurgical phases, if it is the case. Figure 5 shows the temperature and phase dependence of thermal conductivity.

An important feature of the proposed behavior is the transition between either, powder to liquid or liquid to solid. It considers conditions far from the thermodynamic equilibrium associated to the high speed of the heating induced by the laser in the material, and the subsequent cooling, which is something considered in some references for the case of high power laser treatments [20]. In this way, the change of state, instead of being quasi-instantaneous, as for a situation of equilibrium, takes place along a temperature interval following a sigmoid behavior. It is reflected on the thermo-physical properties.

**Figure 6:**
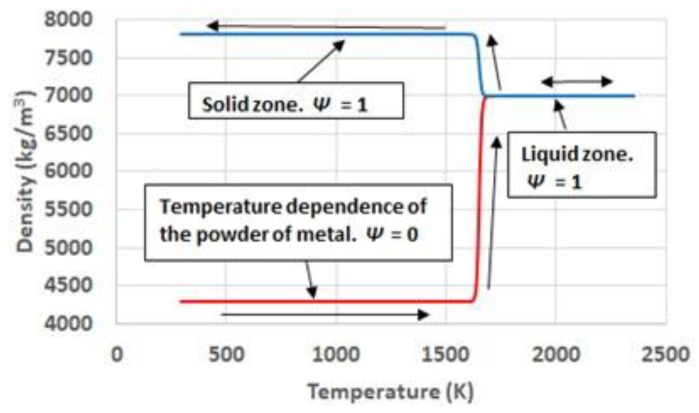
Temperature and phase dependence of density.

Density, considering the same hypotheses as for thermal conductivity, shows an intuitive behavior. It evolves from the apparent density corresponding to the powder of metal, to the density corresponding to the metal liquid. During the cooling, those regions which have molten, use the evolution of density with temperature corresponding to a solid. Figure 6 illustrates the phase and temperature dependence of density. 

Dynamic viscosity plays a crucial role in the evolution of the shape of the material treated. The value of the dynamic viscosity of a liquid steel can be found in [16]. It can be maintained constant along the liquid state. Dynamic viscosity can also be used to emulate the behavior of a solid during the cooling of the material. By associating the dynamic viscosity at room temperature with a value high enough, the shape acquired by the melt pool during the time that the material is in liquid state, can be maintained when it cools. This methodology is proposed by [21] for the keyhole welding process, where a value of 100 Pa·s is considered to be enough to reproduce the behavior of the solid metal. The value of dynamic viscosity to represent the behavior of the powder of metal is the variable surrounded by a higher level of uncertainty. In this work it has been estimated by considering the size and the dimensions of the droplet formed. The formation of it takes place in a volume where the droplet is surrounded by powder. Several experiments have been carried out by using a powder bed with a relatively high thickness of 1 mm. The objective of using such a large layer lies in investigating whether a big volume of powder tends to limit somehow the capability of the melt pool to acquire a free shape. If this is not the case, it can be concluded that powder presents no significant wear for the development of the melt pool, and, therefore, its dynamic viscosity is negligible. The fundamentals of this analysis lie in the comparison of it with the re-melting test where a laser beam is applied on a solid metallic plate [22]. When this is done, despite the material reaching the liquid state, no droplet formation happens. It can be associated with the limitations that the solid metal surrounding the melt pool imposes on the free movement of it.

**Figure 7:**
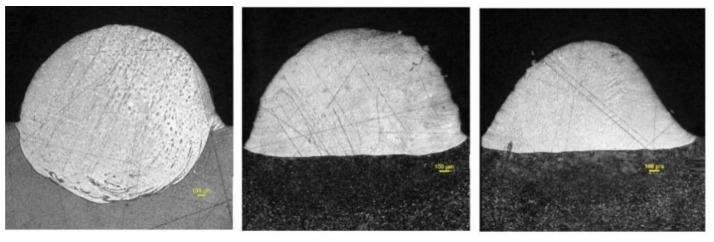
Cross sections of the consolidated material made with a layer of 1 mm of powder with 4000 W, 400 mm/s (**left**), 600 mm/s (**center**) and 800 mm/s (**right**).

Figure 7 contains the image of the cross section of several ribbons obtained after the scanning of the laser beam on a powder layer of 1 mm. In all the cases the power of the laser beam was 4000 W and the scanning speed of 400 mm/s, 600 mm/s and 800 mm/s.

**Figure 8:**
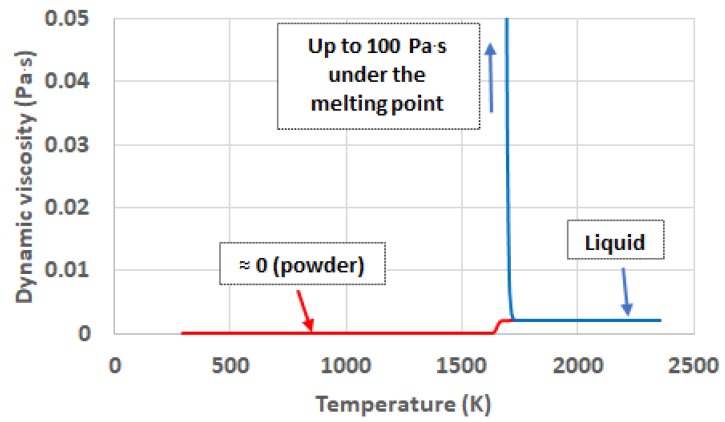
Phase and temperature dependency of dynamic viscosity.

From the results in Figure 8 it can be assumed that the powder around the melt pool, despite having a high thickness, it has not prevented the liquid material from adopting the corresponding drop shape. Consequently, the dynamic viscosity of the metallic powder is considered negligible in comparison with the value of the liquid. Figure 8 contains the proposed phase and temperature functions for dynamic viscosity.

Finally, the surface tension is the last variable that is introduced as critical for the definition of the shape of the melt pool. In the liquid state it shows dependence with temperature as line function with negative slope. The particular value for the working material is reported by [8]. The consideration of this dependence with temperature in the liquid state plays a crucial role in the definition of the melt pool shape, since it is related to the so-called Marangoni Convection. Under the influence of this phenomenon, the liquid metal flows from the regions with low surface tension to the regions where the surfaced tension is higher. In the case of the metal powder, since there is no continuous surface, it can be considered that the surface tension shows a negligible value. For the case of the solid metal the attribution of a value for the surface tension makes no sense. The material is going to be frozen under the effect of a high value of dynamic viscosity and no tangential effects on its surfaces can affect the shape of it. 

**Figure 9:**
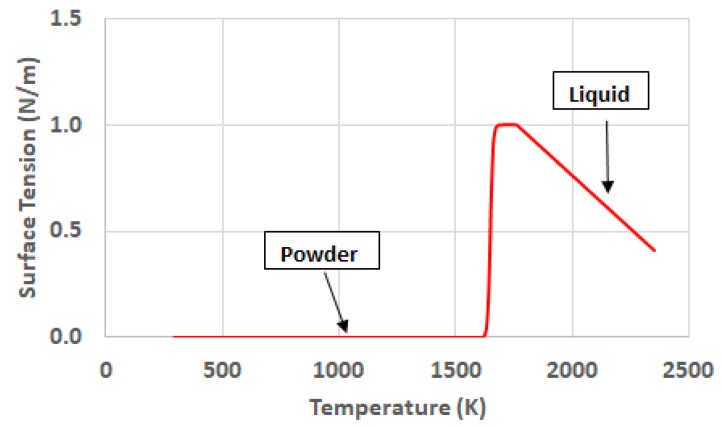
Phase and temperature dependency of the surface tension.

Conclusively, the important aspects of the surface tension are the transition of the material from powder to liquid, and the temperature dependence of it in the liquid state. Figure 9 shows the phase and temperature evolution of the surface tension. Reference [23] highlights the influence of the surface tension in the Selective Laser Melting Process by the effect of the capillarity force on the melt pool.

### 2.3. Typical Output from the Simulation Process

**Figure 10:**
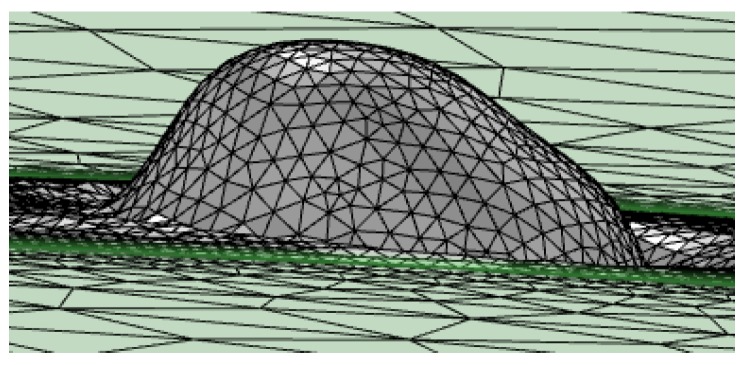
Ribbon formed during the interaction time of the laser beam.

As a result of the modeling process, involving the thermofluidic coupling and the phase and temperature dependency of the of the thermo-physical properties, the development of a consolidated ribbon is obtained. Figure 10 presents the consolidated ribbon obtained during the interaction time of the laser beam, *τ* (*τ = Ø/V*) for a process speed of 600 mm/min and a beam power of 1800 W with a beam diameter of 3 mm. The simulation during the interaction time allows for representing the effect of the full traversing of the laser beam for a given point and analyzing the characteristic dimensions of it, within manageable computation times. The model is also prepared to take into account the recoil pressure [24], although, provisionally it has not been considered for the proposed tests, given the relatively moderated heating conditions and the large amount of material involved.

**Figure 11:**
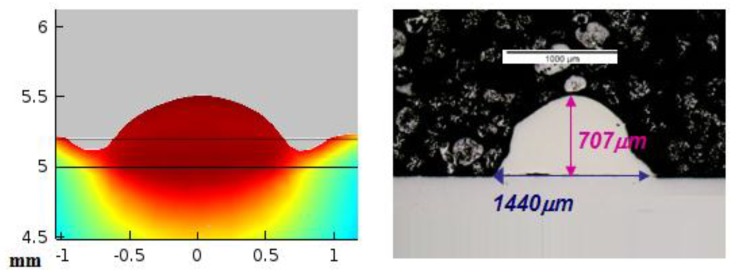
Theoretical (**left**) and experimental (**right**) cross sections.

Figure 11 shows the way in which the results of the simulation are exploited, by comparing the height and width of the cross section predicted by the simulation with the experimental ones.

The next section carries out some representative comparisons between theoretical and experimental results for some tests carried out with typical sets of process parameters. 

## 3. Results

The main goal of the proposed model is the prediction of the shape and dimensions of the cross section of the consolidated material. The progress of the process, in terms of the advance in the direction in which the part is being grown, is determined by the final height of the cross section. In addition, the heat conduction conditions for the consolidation process of the successive layers depend on the geometry of the previously consolidated material, which configures the paths for the diffusion of the heat.

All the tests were made on a substrate layer of 1000 µm, with a gaussian laser beam with a diameter of 3 mm. The laser equipment was a fiber IPG laser with 6 kW of maximum output power releasing radiation with a wavelength of 900 nm.

Three different tests have been carried out. The corresponding conditions of power and scanning speed are shown in Table 2.

**Figure 12:**
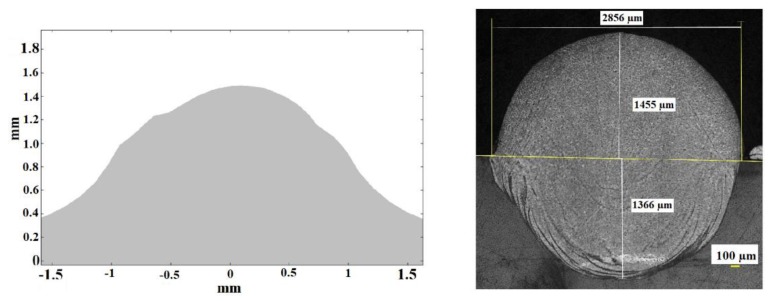
Theoretical (**left**) and experimental (**right**) cross sections corresponding to test 1. Note that in the case of the experimental one the dilution under the substrate is shown while the theoretical one represents exclusively the external part of the consolidated material, from the top of the substrate.

Figure 12 shows the cross section predicted by simulation (left) and the real result obtained in the experimental test number 1 (right). It can be seen that the theoretical calculation has been capable of predicting the height of the consolidated material, as well as the general shape of the area. In both the theoretical and the experimental cross section, the maximum height of the consolidated material, from the reference line of the substrate, lies around 1400 µm (note that the cross section displayed from the theoretical results starts at the level of the substrate, while in the experimental case the dilution under the substrate is shown). In the same way, the width of the cross section at the level of the line of the substrate is of about 2800 µm. The biggest discrepancies between the theoretical and the experimental cross sections appear at the sides of it, at the level of the substrate. It may be associated to dynamic viscosity attributed to the solid in the numerical simulation which predicts a slightly higher level of consistence for the consolidated material than in reality. No contrast of the amount of dilution of the consolidated material into the substrate is highlighted in the numerical simulation, which must be predicted by means of metallurgical consideration from the thermal cycle and will be considered in future developments of the model.

**Figure 13:**
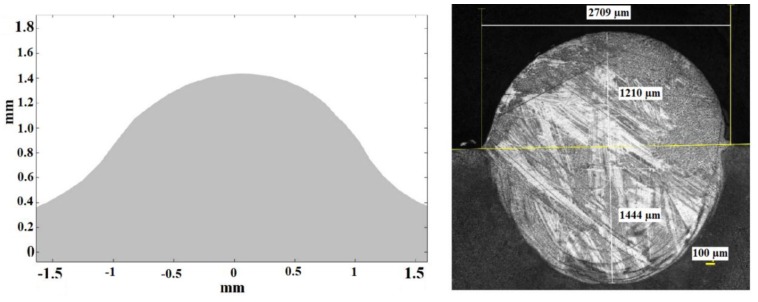
Theoretical (**left**) and experimental (**right**) cross sections corresponding to test 2.

Figure 13 shows the theoretical (left) and experimental (right) cross sections corresponding to test 2.

In Figure 13 it can be seen again, from the cross section of the experimental test, that the high thickness of the layer of metallic powder has given rise to a high dilution of the consolidated material into the substrate. Considering the dimensions of the external part of it, the simulation has been capable of predicting its maximum height and width.

**Figure 14:**
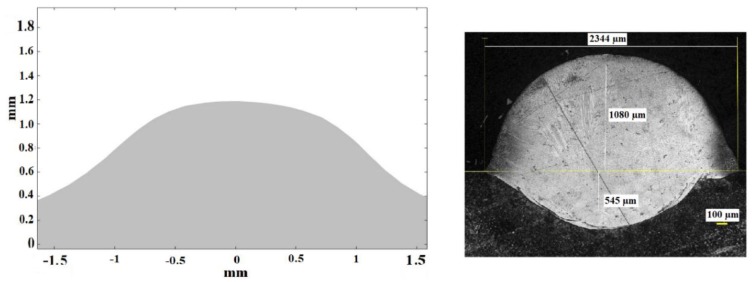
Theoretical (**left**) and experimental (**right**) cross sections corresponding to test 3.

Figure 14 represents the theoretical (left) and experimental (right) results for test 3. Once again, the general external dimensions predicted by the simulation match with an acceptable level of accuracy the dimensions of the experimental cross section. In this case, the use of the highest scanning speed has led to a shorter height of the consolidated material.

## 4. Discussion

The use of temperature and phase-dependent thermo-physical properties has allowed an accurate prediction of the dimensions of the cross section. While the influence of the exclusively thermal properties, such as the thermal conductivity, cannot be directly analyzed; the proposed modelling for the surface tension and the dynamic viscosity is accurate enough to make the output of the simulation approach the experimental results.

From the experimental-theoretical comparison some ideas can be extracted. While the general dimensions of the cross sections can be satisfactorily predicted by the theoretical calculations in terms of height and width, the shape of it, at the level of the contact with the substrate is affected, in the theoretical calculations, by some effect of adhesion that makes the transition from the consolidated material to the substrate smoother than in the real case. It may be associated with the theoretical evolution of the dynamic viscosity which, when it is representing the behavior of the solid, tends to slightly overestimate the adhesion phenomena. Both, numerical calculations and experimental tests highlight the influence of the large thickness of the powder bed in the transformation experienced by the material, evolving from a flat layer of powder to a taller ribbon whose cross section has the shape of a drop. In other papers where a much shorter layer of thickness is considered, this evolution of the material during the drop formation is less evident. Reference [25] is capable of estimating the shape of the melt pool in the SLM process of nanocomposites just by means of thermal calculation. It is possible due to the subtle motion of the liquid metal derived from the small thickness of the powder bed. References [26,27,28] present different SLM process with applications in various fields. The powder bed is not lager than 50 µm in any of the cases. The comparison of the melt pool in the figures of that papers with Figure 12, Figure 13 and Figure 14 of the present study highlights the influence of the thickness of the powder bed in the shape and size of the consolidated material, justifying the use of a thermo-fluidic approach.

From a strictly experimental point of view, the use of a high thickness powder layer is associated to a high degree of dilution of the consolidated material into the substrate. It can be related with the greater amount of material which is melted because of the large thickness of the layer and the size and big power of the laser beam. The inclusion of metallurgical considerations in new versions of the model will focus on calculating the magnitude of the dilution. The aggressiveness of the process parameters used in the present study in comparison with studies with a shorter powder layer can be checked considering the process parameters in the tests of references [29,30].

The relation of the process parameters from the experimental tests with the dimensions of the melt pool, as a function of the thermo-fluidic properties of the material, provides a deep understanding of the physics involved in the consolidation process. In test 1, despite having a relatively low value of laser power, 1000 W, the large interaction time associated to the relatively low process speed, 400 mm/min, has permitted an efficient absorption of the energy to be achieved. In Section 2.1.1 the high efficiency of the powder to absorb radiation was shown. Additionally, in Section 2.2, the poor thermal conductivity of the powder was indicated; it favors the concentration of the heat around the laser-powder interaction area. The combination of a large interaction time with an efficient absorption and concentration of the laser power has led to a melt pool of relatively large dimensions. The hypothesis of considering the powder around the melt pool as a fluid with very low friction (very low dynamic viscosity), can be accepted from the result of this test: the spherical shape of the cross section indicates that the melt pool has not found appreciable resistance to move under the effect of surface tension. No signs of porous in the cross section, which suggests that the density of the material has evolved from the value corresponding to the powder to the value of the solid during the consolidation process.

Test 2 presents a more aggressive combination of process parameters than test 1. The higher level of power, 2000 W, has allowed a larger amount of energy to traverse the powder to the substrate, which, in combination with an intermediate level of process speed, 600 mm/min, has favored a deeper penetration of the melt pool into the substrate. Once again, the low friction of the powder around the melt pool has allowed the external part of it acquire spherical shape. The high degree of symmetry of this contour reinforces the idea of the very limited restriction of the powder to the movement of the melt pool, as considered in the thermo-fluidic coupled model.

In the case of test 3, the use of the most aggressive combination of process parameters highlights the importance of the dynamic effects during the consolidation phenomenon. Despite having the largest level of power, 3000 W, the shorter interaction time has limited both the process of drop formation and the penetration of the melt pool into the substrate. The shape of the external part of the melt pool presents a profile which is not as close to a sphere as the profile of the previous tests: the limited interaction time has led to the cooling of the material before it has been capable of finishing its evolution to a spherical profile. In addition, the heating and cooling processes, happening more quickly than in the previous tests, limits the time of the molecules of the material to move from their equilibrium positions, restricting any diffusion process which might occur as a consequence of the thermal cycle.

The numerical aspects of the theoretical model have allowed the convergence of the calculations to be achieved. The mesh deformed under the effect of the loads affecting the fluid, and automatically re-meshed when the distortion of the elements reached the user-defined maximum permitted value.

## 5. Conclusions

The thermo-fluidic coupling has been revealed as a valid tool to evaluate the behavior of the material in its evolution from powder to liquid and from liquid to solid. The dimensions of the cross section predicted by the simulations matched the experimental results with an acceptable level of accuracy. It reinforces the idea of phase and temperature-dependent thermo-physical properties to understand the behavior of the material during the different stages that it experiences in the Selective Laser Melting Process. 

The use of the finite element method in combination with advanced numerical tools has allowed realistic domains with large dimensions to be used, thus favoring, in this way, the study or real processes at an industrial scale, surpassing the capabilities of conventional models with domains at the microscale level.

The governability of the process with a large thickness powder bed has been revealed to be possible, since the simulations have been capable of estimating the dimensions of the cross section resulting from a particular combination of process parameters. Specific consideration about the amount of dilution associated with large powder beds are necessary. Future works dealing with metallurgical aspects of the process will be carried out. 

## Figures and Tables

**Table 1:** Composition and particle size distribution of the MetcoClad™ 316L-SI powder.

Composition (%wt)	Fe	Ni	Cr	Mo	Si	Mn	C	Others
	Balance	12.0	17.0	2.5	2.3	1.0	0.03	≤0.05
Particles size (µm)	Nominal Range		--	(%) > 106	44 < (%) < 106		(%) < 44	
	44–106		--	5	90		5	

**Table 2:** Conditions of the tests to compare the theoretical with the experimental results.

Test Number	Laser Power (W)	Scanning Speed (mm/min)
1	1000	400
2	2000	600
3	3000	800
